# Mixed Convection Hybrid Nanofluid Flow Induced by an Inclined Cylinder with Lorentz Forces

**DOI:** 10.3390/mi14050982

**Published:** 2023-04-29

**Authors:** Farizza Haniem Sohut, Umair Khan, Anuar Ishak, Siti Khuzaimah Soid, Iskandar Waini

**Affiliations:** 1 Department of Mathematical Sciences, Faculty of Science and Technology, Universiti Kebangsaan Malaysia, Bangi 43600, Malaysia; p106827@siswa.ukm.edu.my (F.H.S.); umairkhan@iba-suk.edu.pk (U.K.); 2 Department of Mathematics and Social Sciences, Sukkur IBA University, Sukkur 65200, Pakistan; 3 School of Mathematical Sciences, College of Computing, Informatics and Media, Universiti Teknologi MARA, Shah Alam 40450, Malaysia; khuzaimah@tmsk.uitm.edu.my; 4 Faculty of Mechanical and Manufacturing Engineering Technology, Universiti Teknikal Malaysia Melaka, Hang Tuah Jaya, Durian Tunggal, Melaka 76100, Malaysia; iskandarwaini@utem.edu.my

**Keywords:** magnetohydrodynamic (MHD), mixed convection, inclined cylinder, stagnation point, hybrid nanofluid, dual solutions, stability analysis

## Abstract

Hybrid nanofluids may exhibit higher thermal conductivity, chemical stability, mechanical resistance and physical strength compared to regular nanofluids. Our aim in this study is to investigate the flow of a water-based alumina-copper hybrid nanofluid in an inclined cylinder with the impact of buoyancy force and a magnetic field. The governing partial differential equations (PDEs) are transformed into a set of similarity ordinary differential equations (ODEs) using a dimensionless set of variables, and then solved numerically using the bvp4c package from MATLAB software. Two solutions exist for both buoyancy opposing (λ < 0) and assisting (λ > 0) flows, whereas a unique solution is found when the buoyancy force is absent (λ = 0). In addition, the impacts of the dimensionless parameters, such as curvature parameter, volume fraction of nanoparticles, inclination angle, mixed convention parameter, and magnetic parameter are analyzed. The results of this study compare well with previously published results. Compared to pure base fluid and regular nanofluid, hybrid nanofluid reduces drag and transfers heat more efficiently.

## 1. Introduction

A nanofluid is a solid-liquid mixture containing nanoparticles and a base liquid. According to Daungthongsuk and Wongwises [1], the thermal conductivity of a fluid is boosted by mixing nanoparticles with a regular fluid. Because thermal conductivity is the most important issue in heat transfer problems, many mathematical models for nanofluids have been developed by researchers such as Buongiorno [2] and Tiwari and Das [3]. In addition, issues surrounding the improvement of heat transfer are also important for other cases such as employing microchannel heat sink (MCHS) with V-ribs and electrohydrodynamic (EHD) using water-based nanofluid. The heat transfer performance of a MCHS is enhanced due to the periodically arranged V-ribs that interrupt the thermal boundary, which produce chaotic convention and augment the heat transfer area [4]. Wang et al. [5] mentioned that the major processes involved in increasing heat transfer in microchannels are chaotic convection caused by the electrophoretic effect and thermophoretic effect. Although nanofluids have extremely efficient qualities and properties for industrial and engineering operations, researchers are still searching for fluid variants by studying several of their physical aspects such as the type, shape, and volume fraction of nanoparticles. Hybrid nanofluid exhibits significant developments in its thermal and rheological properties when compared to regular nanofluid, particularly in improving the heat conductivity of base fluids. The upgraded features of nanofluids result from the addition of two or more types of nanoparticles to a common fluid that is called a hybrid nanofluid and serves the purpose of increasing the heat transfer rate. Suresh et al. [6] mentioned that researchers have used a variety of nanoparticles including carbon nanotubes, metallic particles (Cu, Al, Fe, Au, and Ag), and non-metallic particles (Al_2_O_3_, CuO, Fe_3_O_4_, TiO_2_, and SiC). Furthermore, if the nanoparticle materials are properly chosen, they can positively enhance each other and significantly improve the thermal conductivity of the nanofluid. Based on the characteristics of metallic and non-metallic nanoparticles, it can be expected that the addition of metallic nanoparticles such as copper (Cu) into a nanofluid composed on a basis of Al_2_O_3_ nanoparticles can enhance the thermophysical properties of this mixture. It has been determined that most hybrid nanofluids studied have a higher thermal conductivity than nanofluid, but Guan et al. [7] further explored the reason why hybrid nanofluids have high thermal conductivity. The nanolayer densities and diffusion coefficients were calculated for various hybrid nanofluids to explain the underlying mechanism of the enhancement in the thermal properties. The results showed that a hybrid nanofluid with Cu-Ag 50%/Ar can obtain the greatest improvement in thermal conductivity compared to liquid Ar, Cu/Ar and Ag/Ar nanofluids.

Waini et al. [8] reported that a hybrid nanofluid, also known as a base fluid with multiple types of nanoparticles, had improved thermal properties. Highly effective thermal conductivity, enhanced heat transfer, the synergistic effect of nanoparticles, stability, and good aspect ratio are the advantages of a hybrid nanofluid. Lower operating costs and higher energy efficiency are the best definitions of enhanced thermal conductivity of a hybrid nanofluid [9]. A hybrid nanofluid mixed convection flow has applications in various modern manufacturing processes, such as metal extrusion, wire drawing, welding, and heat pipe, etc. [10]. Suresh et al. [11] investigated how an Al_2_O_3_-Cu/H_2_O hybrid nanofluid affected heat transfer. They identified that the hybrid nanofluid had a greater friction coefficient than that of an Al_2_O_3_/H_2_O nanofluid. Alshuhail et al. [12] reviewed the thermal efficiency of hybrid nanofluids in solar energy applications. Ranga et al. [13] reported that for Cu-CuO/H_2_O hybrid nanofluid at 5% volume fraction, the collector efficiency increased by 2.175%. An experimental analysis conducted by Farajzadeh et al. [14] found that the collector efficiency increased by 26% on using Al_2_O_3_-TiO_2_/H_2_O hybrid nanofluids.

The mathematical analysis of the dual solutions of the boundary layer flow along moving surfaces has a practical application in the engineering scenario. It enables the determination of the most practical, stable, and physically acceptable solutions. Early research on Blasius flow for non-unique solutions by considering various flow situations and boundary conditions was conducted by Steinheuer [15]. He performed an investigation on boundary layer flow over a semi-infinite long flat plate with moving but impermeable soil to give a non-unique solution. Later, Klemp and Acrivos [16] reported that non-unique solutions exist in the case where the plate and the free stream move with the reverse flow. The flow field differs from the ordinary boundary layer in that an inviscid collision area exists around the site of detachment, where the reverse boundary-layer flow is turned in the direction of the mainstream. In addition, other scholars such as Bognár [17], Hussaini and Lakin [18], and Ishak and Bachok [19] reported similar problems with the moving flat plate for Newtonian fluid flow. It was discovered that the existence of solutions for a semi-infinite plate is dependent on the ratio of the plate surface velocity to the free stream velocity. It has been demonstrated that a solution exists only if this parameter does not exceed a specific critical value, and numerical computations have been performed to demonstrate that this solution is non-unique. However, the existence of non-unique solutions also occurs for the Blasius boundary conditions problem when the surface is at rest, as reported by Khashi’ie et al. [20], Waini et al. [21], Sohut et al. [22], etc.

A stagnation point (SP) is a point on a solid body which directly faces the stream and where the streamlines are separated. On the other hand, the flow of a fluid in the neighborhood of an SP is called stagnation point flow (SPF). The exploration of SPF is a hot topic in fluid mechanics because of its functions and applications in industries. The stagnation point flow, also commonly known as the Hiemenz flow, was established by Hiemenz [23] and can be accurately analyzed using the Navier–Stokes equation. He was the first researcher to find an outcome for the two-dimensional SPF model over a horizontal plate. Hiemenz [23] solved the Navier–Stokes equation numerically by transforming it into a regular differential equation using a suitable similarity transformation. Many cases that involved SPF have also been discovered by many scholars. For instance, Chiam [24] explored the SPF past a stretching sheet. Wang [25] examined both axisymmetric and two-dimensional stagnation flows toward a shrinking sheet. He found that the flow structure is complicated due to the non-alignment of the stagnation flow on the shrinking sheet. A few years later, Lok et al. [26] continued the investigation by including the effect of MHD. However, due to the shrinkage effect, the streamlines are not always parallel, but a reverse flow is designed or originated near the surface. Furthermore, Awaludin et al. [27] investigated the SPF over a shrinking as well as a stretching sheet. They found that the solution for the stretching case is unique, but two solutions were found for the shrinking case.

Many industrial applications and natural phenomena involve mixed convection flows (MCFs), which involve a combination of free and forced convection flows, such as electronic equipment cooled by fans, flows in the ocean, underground cable systems, etc. In MCF, the existence of dual solutions has recently become a subject of discussion among scholars. For example, Ishak et al. [28] explored the magnetohydrodynamic MCF on a vertical continuous porous wall using a finite difference method. They reported that both opposing and assisting flows produce dual solutions. The existence of the solutions is dependent on the buoyancy parameter, where the range increases with suction effect at the boundary. Later, Aman et al. [29] continued the study by adding the slip effect at the boundary. Abbas et al. [30] studied the MHD mixed convection flow between concentric cylinders and reported that the mixed convection increases the velocity on the heated cylinder, but it decreases on the cold cylinder. The combined effects of MHD and slip velocity on the mixed convection flow around a circular cylinder was investigated by Ullah et al. [31]. They reported that an increase in slip factor enhances velocity as well as temperature. The MHD flow of a hybrid nanofluid past a vertical plate was considered by Zainal et al. [32], who found two branches of solutions in a certain range of the mixed convection strength. The temporal stability study verified that only one of the solutions was stable. Wang et al. [33] explored the influence of magnetic and electric fields on heat transfer to analyze the thermal conductivity and heat transfer enhancement of nanofluids, respectively, and the chaotic convention. They found that the applied magnetic and electric fields significantly affect the heat transfer performance of the nanofluid.

The study of fluid flow over an inclined cylindrical surface has significance for applications in industrial and engineering processes, e.g., magnetohydrodynamic (MHD) power generators, the polymer industry, and gas turbines [34]. Studies on the flow over a horizontal or a vertical cylinder have been explored widely; however, studies involving an inclined cylinder remain uncommon. An analysis of stratified stagnation flow induced by an inclined cylinder with mixed convection and a magnetic field was presented by Hayat et al. [35]. Bilal et al. [36] studied the Darcy Forchheimer flow of a hybrid nanofluid (consisting of carbon nanotubes) through the impermeable inclined cylinder using a homotopy analysis method. They found that the higher the inclination angle, the slower the fluid flow. Rehman et al. [37] extended the study to include logarithmic and parabolic curve fitting analysis and stated that analysis is the first step and can be of great help to previous studies. In addition, the study of MHD mixed convection on an inclined cylinder has also attracted the attention of researchers for the case of nanofluid flow, such as Dhanai et al. [38], who examined the effects of slip boundary conditions, Brownian motion, thermophoresis, and viscous dissipation over an inclined cylinder. They discovered that heat transfer increases with the augmentation of the mass transfer parameter but the opposite occurs for the thermal slip parameter. Moreover, Gupta and Sharma [39] studied a similar problem in the presence of thermal radiation. They used the differential transform method (DTM) with Padé approximation in their study and found that the DTM can overcome traditional perturbation limits, assumptions and restrictions.

Based on the previous investigations, hybrid nanofluid flow over an inclined cylinder has not been extensively studied. Binary hybrid nanofluid flow is a popular topic in fluid dynamics inspired by the above literature. The current investigation intends to discover and analyze the impact of magnetohydrodynamic on hybrid nanofluid flow over an inclined cylinder. This study is the extension of what has been investigated by researchers, such as Rehman et al. [37] and Devi and Devi [9], which considers binary hybrid Al_2_O_3_-Cu/H_2_O nanofluid. The stimulus of the governing parameters is generally represented via several graphs and tables, and the quantitative outcomes obtained are validated for the limited cases using the results of previous studies. Furthermore, the current study reveals that the stability investigation proves the physical outcome. These duality and stability results were also the main objectives of the current work.

## 2. Description of the Mathematical Model

**Figure 1:**
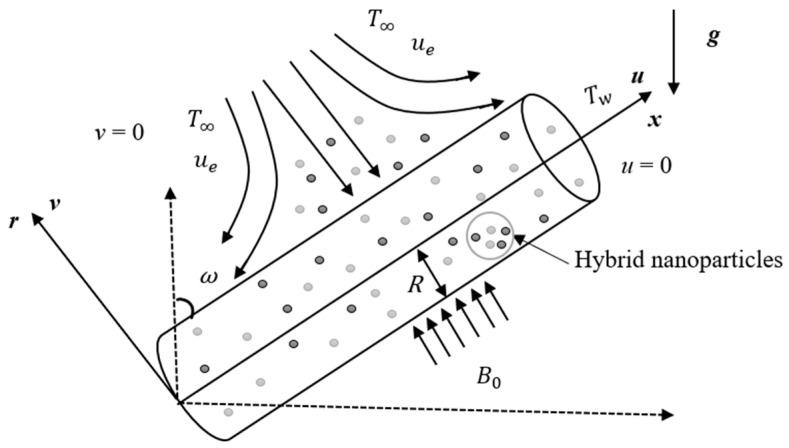
Physical configuration.

Consider the buoyancy effects on a magnetohydrodynamic SPF of a steady incompressible hybrid nanofluid past an inclined cylindrical surface, as shown in Figure 1. The coordinates $\left(x,r\right)$ are measured in the corresponding axial and radial directions of the cylinder. It is assumed that the temperature of the cylindrical surface, $T_{w}$, is higher than the surrounding temperature, $T_{\infty }$. In the current study, the thermophysical properties of the hybrid nanofluid are taken to be uniform. Meanwhile, copper nanoparticles are combined with the base fluid to produce Cu/H_2_O nanofluid, which is then combined with the alumina nanoparticles, Al_2_O_3_, to create the required hybrid nanofluid, Al_2_O_3_-Cu/H_2_O.

The boundary layer equations comprising hybrid nanofluid can take place as follows [9,37]:

(1) $$\frac{\partial \left(ru\right)}{\partial x}+\frac{\partial \left(rv\right)}{\partial r}=0$$

(2) $$\begin{array}{l} \rho_{hnf}\left(u\frac{\partial u}{\partial x}+v\frac{\partial u}{\partial r}-u_{e}\frac{\partial u_{e}}{\partial x}\right)=\mu_{hnf}\frac{1}{r}\frac{\partial }{\partial r}\left(r\frac{\partial u}{\partial r}\right)-\sigma_{hnf}B_{0}{}^{2}\left(u-u_{e}\right)\\ +{\left(\rho \beta \right)}_{hnf}\left(T-T_{\infty }\right)g\cos\omega \end{array}$$

(3) $${\left(\rho C_{p}\right)}_{hnf}\left(u\frac{\partial T}{\partial x}+v\frac{\partial T}{\partial r}\right)=k_{hnf}\frac{1}{r}\frac{\partial }{\partial r}\left(r\frac{\partial T}{\partial r}\right)$$

subject to the boundary conditions

(4) $$\begin{array}{l} \;r=R:\;\;\;\;u=0,\;\;\;\;v=0,\;\;\;\;T=T_{w},\;\\ \;r\to \infty :\;\;\;\;u\to u_{e}=ax,\;\;\;\;T\to T_{\infty }.\; \end{array}$$

Here, v and u are the elements of velocity along the r and x axes, respectively, $B_{0}$ is the uniform magnetic field, g is the gravity acceleration, ω is the inclination angle, T is the temperature of the hybrid nanofluid, and a is a positive constant.

Where

(5) $$\mu_{H}=\frac{\mu_{hnf}}{\mu_{f}},\;\rho_{H}=\frac{\rho_{hnf}}{\rho_{f}},\;\sigma_{H}=\frac{\sigma_{hnf}}{\sigma_{f}},\;k_{H}=\frac{k_{hnf}}{k_{f}},\;{\left(\rho C_{p}\right)}_{H}=\frac{{\left(\rho C_{p}\right)}_{hnf}}{{\left(\rho C_{p}\right)}_{f}},\;{\left(\rho \beta \right)}_{H}=\frac{{\left(\rho \beta \right)}_{hnf}}{{\left(\rho \beta \right)}_{f}}$$

In Table 1, $\phi_{hnf}=\phi_{1}+\phi_{2}$ is the hybrid nanoparticle volume fraction, where $\phi_{f}$ corresponds to the regular liquid. In addition, $\phi_{1}$ and $\phi_{2}$ indicate aluminum oxide (Al_2_O_3_) and copper (Cu) nanoparticle volume fractions, and their solid components are symbolized by the subscripts Al and C, respectively. In addition, μ, ρ, k, $C_{p}$, σ, and β refer to the absolute viscosity, density, TCN, specific heat capacity, electrical conductivity and thermal expansion coefficient, respectively. The subscripts *hnf* and *f*, respectively, stand for the requisite posited hybrid nanofluid and the regular fluid. Table 2 provides the values of the thermophysical characteristics of the nanoparticles and water (regular fluid).

In order to get similarity solutions, the dimensionless variables (6) are introduced [37,44]:

(6) $$\psi ={\left(u_{e}v_{f}x\right)}^{1/2}Rf\left(\eta \right),\;\;\;\;\;\;\eta =\frac{r^{2}-R^{2}}{2R}\sqrt{\frac{u_{e}}{v_{f}x}},\;\;\;\;\;\;\theta \left(\eta \right)=\frac{T-T_{\infty }}{T_{w}-T_{\infty }}$$

where prime denotes differentiation with respect to η, and ψ is the stream function defined as $u=r^{-1}\partial \psi /\partial r$ and $v=-r^{-1}\partial \psi /\partial x$, which identically satisfies the continuity Equation (1). The temperature at the surface of the cylinder is assumed to be $T_{w}=T_{\infty }+bx$, where b is a constant.

By utilizing the similarity variables (6), Equation (1) is identically satisfied, whereas Equations (2)–(4) are transformed into the following ODEs:

(7) $$\frac{\mu_{H}}{\rho_{H}}\left[\left(1+2K\eta \right)f^{\prime \prime \prime }+2Kf^{\prime \prime }\right]+ff^{\prime \prime }+1-{f^{\prime }}^{2}-\frac{\sigma_{H}}{\rho_{H}}M^{2}\left(f^{\prime }-1\right)+\frac{{\left(\rho \beta \right)}_{H}}{\rho_{H}}\lambda \theta \cos\omega =0$$

(8) $$\frac{1}{\mathrm{Pr}}\frac{k_{H}}{{\left(\rho C_{p}\right)}_{H}}\left[\left(1+2\eta K\right)\theta^{\prime \prime }+2K\theta^{\prime }\right]+\theta^{\prime }f-\theta f^{\prime }=0$$

(9) $$\begin{array}{l} f\left(0\right)=0,\;\;\;\;\;\;f^{\prime }\left(0\right)=0,\;\;\;\;\;\;\theta \left(0\right)=1\\ \;f^{\prime }\left(\eta \right)\to 1,\;\;\;\;\;\;\theta \left(\eta \right)\to 0\;\;\;\;\;\;\mathrm{as}\;\;\;\;\;\;\;\eta \to \infty \end{array}$$

where *M* stands for magnetic parameter, Pr is Prandtl number, *K* is the curvature parameter, and λ is the mixed convection parameter (constant). Further, the buoyancy term is equal to the ratio of the local Grashof number, *Gr_x_*, and the square of the local Reynolds number, Rex2. The dimensionless quantities are defined as:

(10) M=σfβ02ρfa,    Pr=vfαf,    K=1Rνfa ,    λ=GrxRex2(=const.),    Grx=gβfTw−T∞x3νf2

The skin friction coefficient and local Nusselt number (heat transfer rate) are given by:

(11) $$C_{f}=\frac{\mu_{hnf}}{\rho_{f}u_{e}{}^{2}\left(x\right)}{\left(\frac{\partial u}{\partial r}\right)}_{r=R},\;\;\;\;\;\;Nu_{x}=-\frac{xk_{hnf}}{k_{f}\left(T_{f}-T_{\infty }\right)}{\left(\frac{\partial T}{\partial r}\right)}_{r=R}$$

which, after applying (5) and (11), then become the following:

(12) Rex1/2Cf=μHf″0,      Rex−1/2Nux=−kHθ′0

where Rex=uex/vf is the local Reynolds number.

## 3. Stability Analysis

Several researchers [45,46,47] have demonstrated the temporal stability of the numerical solutions. The purpose being to verify the stability of the solutions as time evolves. In the present study, two solutions for Equations (7)–(9) are found within a certain range of the physical parameters. It is worth investigating the time stability of the solutions and determining which one of the two solutions is reliable, and which one is not reliable, over time. For the working procedure, the following dimensionless time variable τ=at is introduced, as proposed by Merkin [48] and Weidman et al. [49]. The unsteady or time-dependent form of Equations (2) and (3) are considered, and then they are reduced to:

(13) $$\begin{array}{l} \rho_{hnf}\left(\frac{\partial u}{\partial t}+u\frac{\partial u}{\partial x}+v\frac{\partial u}{\partial r}-u_{e}\frac{\partial u_{e}}{\partial x}\right)=\mu_{hnf}\frac{1}{r}\frac{\partial }{\partial r}\left(r\frac{\partial u}{\partial r}\right)-\sigma_{hnf}B_{0}{}^{2}\left(u-u_{e}\right)\\ +{\left(\rho \beta \right)}_{hnf}\left(T-T_{\infty }\right)g\cos\omega \end{array}$$

(14) $${\left(\rho C_{p}\right)}_{hnf}\left(\frac{\partial T}{\partial t}+u\frac{\partial T}{\partial x}+v\frac{\partial T}{\partial r}\right)=k_{hnf}\frac{1}{r}\frac{\partial }{\partial r}\left(r\frac{\partial T}{\partial r}\right)$$

where *t* stands for time. Based on Equation (6), the dimensionless transformations are written as follows:

(15) $$\begin{array}{l} \psi ={\left(u_{e}v_{f}x\right)}^{1/2}Rf\left(\eta ,\tau \right),\;\;\;\;\;\eta =\frac{r^{2}-R^{2}}{2R}\sqrt{\frac{u_{e}}{v_{f}x}},\;\;\;\;\;u=ax\;f^{\prime }\left(\eta ,\tau \right),\\ v=-\frac{R}{r}\sqrt{av_{f}}f\left(\eta ,\tau \right),\;\;\;\;\;\theta \left(\eta ,\tau \right)=\frac{T-T_{\infty }}{T_{w}-T_{\infty }}. \end{array}$$

Substituting Equation (15) into Equations (13) and (14) results in

(16) $$\begin{array}{l} \frac{\mu_{H}}{\rho_{H}}\left[\left(1+2K\eta \right)\frac{\partial^{3}f}{\partial \eta^{3}}+2K\frac{\partial^{2}f}{\partial \eta^{2}}\right]-\frac{\partial^{2}f}{\partial \eta \partial \tau }+1+f\frac{\partial^{2}f}{\partial \eta^{2}}-\frac{\sigma_{H}}{\rho_{H}}M^{2}\left(\frac{\partial f}{\partial \eta }-1\right)-{\left(\frac{\partial f}{\partial \eta }\right)}^{2}\\ +\frac{{\left(\rho \beta \right)}_{H}}{\rho_{H}}\lambda \theta \cos\omega =0, \end{array}$$

(17) $$\frac{1}{\mathrm{Pr}}\frac{k_{H}}{{\left(\rho C_{p}\right)}_{H}}\left[\left(1+2K\eta \right)\frac{\partial^{2}\theta }{\partial \eta^{2}}+2K\frac{\partial \theta }{\partial \eta }\right]-\frac{\partial \theta }{\partial \tau }+\frac{\partial \theta }{\partial \eta }f-\frac{\partial f}{\partial \eta }\theta =0,$$

subjected to:

(18) $$\begin{array}{l} f\left(0,\tau \right)=0,\;\;\;\;\;\;\frac{\partial f}{\partial \eta }\left(0,\tau \right)=0,\;\;\;\;\;\;\theta \left(0,\tau \right)=1\\ \frac{\partial f}{\partial \eta }\left(\eta ,\tau \right)\to 1,\;\;\;\;\;\;\theta \left(\eta ,\tau \right)\to 0\;\;\;\;\;\;\;\;\;\;\;\;\mathrm{as}\;\;\;\;\;\;\eta \to \infty . \end{array}$$

To verify the temporal stability of the dual solutions, we follow Weidman et al. [49] by introducing the following perturbations:

(19) $$f\left(\eta ,\tau \right)=f_{0}\left(\eta \right)+e^{-\gamma \tau }F\left(\eta ,\tau \right),\;\;\;\;\;\;\theta \left(\eta ,\tau \right)=\theta_{0}\left(\eta \right)+e^{-\gamma \tau }G\left(\eta ,\tau \right).$$

Here, the functions F and G are considered small compared to the steady solutions $f_{0}$ and $\theta_{0}$, and *γ* stands for an unknown eigenvalue. Substituting (19) into Equations (16)–(18) results in

(20) $$\begin{array}{l} \frac{\mu_{H}}{\rho_{H}}\left[\left(1+2K\eta \right)\frac{\partial^{3}F}{\partial \eta^{3}}+2K\frac{\partial^{2}F}{\partial \eta^{2}}\right]+f_{0}\frac{\partial^{2}F}{\partial \eta^{2}}+F\frac{\partial^{2}f_{0}}{\partial \eta^{2}}+\left(\gamma -2\frac{\partial f_{0}}{\partial \eta }\right)\frac{\partial F}{\partial \eta }-\frac{\sigma_{H}}{\rho_{H}}M^{2}\frac{\partial F}{\partial \eta }\\ +\frac{{\left(\rho \beta \right)}_{H}}{\rho_{H}}\lambda G\cos\omega =0, \end{array}$$

(21) $$\frac{1}{\mathrm{Pr}}\frac{k_{H}}{{\left(\rho C_{p}\right)}_{H}}\left[\left(1+2K\eta \right)\frac{\partial^{2}G}{\partial \eta^{2}}+2K\frac{\partial G}{\partial \eta }\right]-\theta_{0}\frac{\partial F}{\partial \eta }+f_{0}\frac{\partial G}{\partial \eta }+F\frac{\partial \theta_{0}}{\partial \eta }+G\left(\gamma -\frac{\partial f_{0}}{\partial \eta }\right)=0,$$

(22) $$\begin{array}{l} F\left(0,\tau \right)=0,\;\;\;\;\;\;\frac{\partial F}{\partial \eta }\left(0,\tau \right)=0,\;\;\;\;\;\;G\left(0,\tau \right)=0,\\ \;\frac{\partial F}{\partial \eta }\left(\eta ,\tau \right)\to 0\;\;\;\;\;\;G\left(\eta ,\tau \right)\to 0\;\;\;\;\;\;\;\;\;\;\;\;\mathrm{as}\;\;\;\;\;\;\eta \to \infty . \end{array}$$

By rendering τ=0, the stability of the steady flow solutions $f_{0}$ and $\theta_{0}$ can be investigated, and hence the initial decay or growth of the solutions can be identified by $F=F_{0}\left(\eta \right)$ and $G=G_{0}\left(\eta \right)$, respectively. The linearized eigenvalue equations are:

(23) $$\begin{array}{l} \frac{\mu_{H}}{\rho_{H}}\left[\left(1+2K\eta \right)F_{0}{}^{\prime \prime \prime }+2KF_{0}{}^{\prime \prime }\right]+f_{0}F_{0}{}^{\prime \prime }+F_{0}f_{0}{}^{\prime \prime }+\left(\gamma -2f_{0}{}^{\prime }\right)F_{0}{}^{\prime }-\frac{\sigma_{H}}{\rho_{H}}M^{2}F_{0}{}^{\prime }\\ +\frac{{\left(\rho \beta \right)}_{H}}{\rho_{H}}\lambda G_{0}\cos\omega =0, \end{array}$$

(24) $$\frac{1}{\mathrm{Pr}}\frac{k_{H}}{{\left(\rho C_{p}\right)}_{H}}\left[\left(1+2K\eta \right)G_{0}{}^{\prime \prime }+2KG_{0}{}^{\prime }\right]-\theta_{0}F_{0}{}^{\prime }+f_{0}G_{0}{}^{\prime }+F_{0}\theta_{0}{}^{\prime }+G_{0}\left(\gamma -f_{0}{}^{\prime }\right)=0,$$

subject to the boundary conditions

(25) $$\begin{array}{l} F_{0}\left(0\right)=0,\;\;\;\;\;\;F_{0}{}^{\prime }\left(0\right)=0,\;\;\;\;\;\;G_{0}\left(0\right)=0,\\ F_{0}{}^{\prime }\left(\eta \right)\to 0\;\;\;\;\;\;G_{0}\left(\eta \right)\to 0\;\;\;\;\;\;\;\;\;\;\;\;\mathrm{as}\;\;\;\;\;\;\eta \to \infty . \end{array}$$

Following Harris et al. [50], the condition ${F^{\prime }}_{0}\left(\eta \right)\to 0$ as η→∞ in Equation (25) is relaxed, and is substituted by the new boundary condition ${F^{\prime \prime }}_{0}\left(0\right)=1$, without loss of generality. This replacement is performed in order to obtain the smallest eigenvalue $\gamma_{1}$ from the infinite set of eigenvalues $\gamma_{1}<\gamma_{2}<\gamma_{3}\ldots$. The flow solution is considered stable only when the generated smallest eigenvalue is positive $\left(\gamma_{1}>0\right)$, and this positive smallest eigenvalue eventually approaches zero as $\lambda \to \lambda_{c}$, indicating that the solution is stable as the applied perturbation decreases with time.

## 4. Results and Discussion

The system of Equations (7)–(9) was numerically solved using the bvp4c package available from MATLAB R2021a software. It is necessary to set an initial guess at the initial mesh point, and to set the step size, Δ*η*, in order to obtain the specified accuracy. In the present study, we set the boundary layer thickness as $\eta_{\infty }=10$ and the step size as Δη=0.01 with the relative error tolerance set to 10^−10^. The initial guesses are dependent on the parameter values applied for computation. The validity of the numerical results was checked by examining the related profiles. Moreover, the numerical solutions were compared with the available results for the particular case of this study.

Several representing values of parameters were selected in such a way as to examine the behaviors of the heat transport and flow dynamics characteristics. For instance, the present problem comprised several influential parameters such as curvature parameter *K*, mixed convection parameter λ, magnetic parameter *M*, inclination angle ω, and the solid volume fraction of nanoparticles $\phi_{1},\;\phi_{2}$. For clarity, subscripts 1 and 2, which represent Al_2_O_3_ and Cu, respectively, were introduced. In addition, the effects of these parameters on fluid motion, skin friction coefficient, temperature and heat transfer profiles are shown in several graphs and tables. For the purpose of the computations, we used the following default values: *M* = 0.1, *ω* = 30°, Pr = 6.2, *K* = 0.2 and *ϕ*_1_ = *ϕ*_2_ = 0.01. Furthermore, the values of $f^{\prime \prime }(0)$ and $-\theta^{\prime }\left(0\right)$, which signify the skin friction coefficient and the heat transfer rate, were compared with those obtained previously by Lok et al. [51] and Ishak et al. [28], which show a good agreement. This validation is highlighted for the case of assisting flow, λ=1 in the absence of hybrid nanoparticles *ϕ*_1_ = *ϕ*_2_ = 0, curvature parameter *K* = 0, inclination angle *ω* = 0, and magnetic parameter *M* = 0, for different values of Prandtl number Pr, as presented in Table 3 and Table 4.

**Figure 2:**
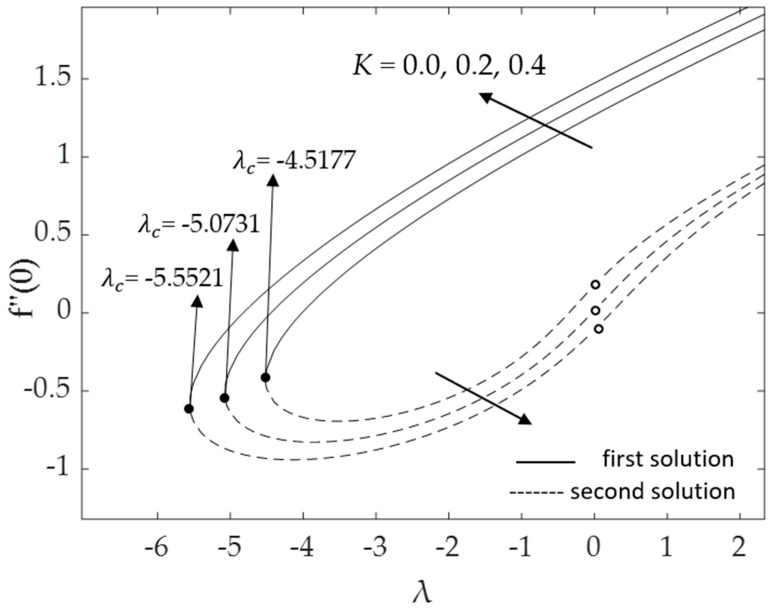
Deviations of $f^{\prime \prime }(0)$ for distinct values of K.

**Figure 3:**
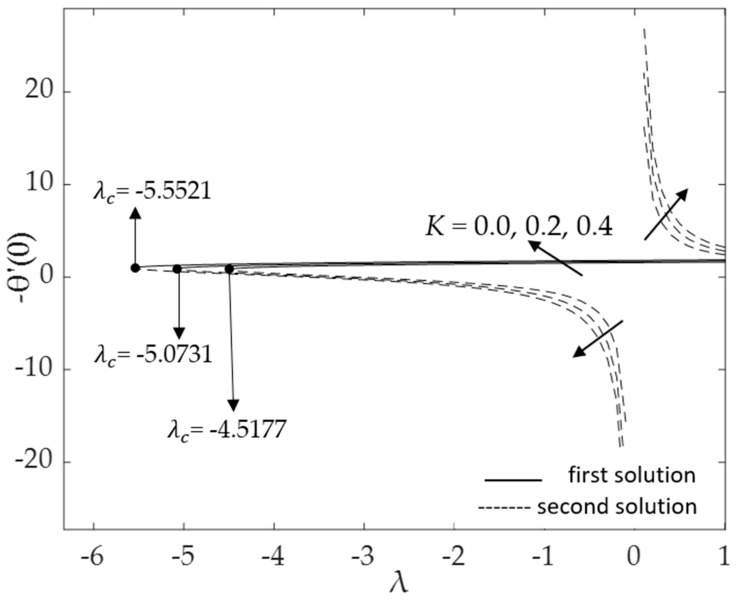
Deviations of $-\theta^{\prime }(0)$ for distinct values of *K*.

**Figure 4:**
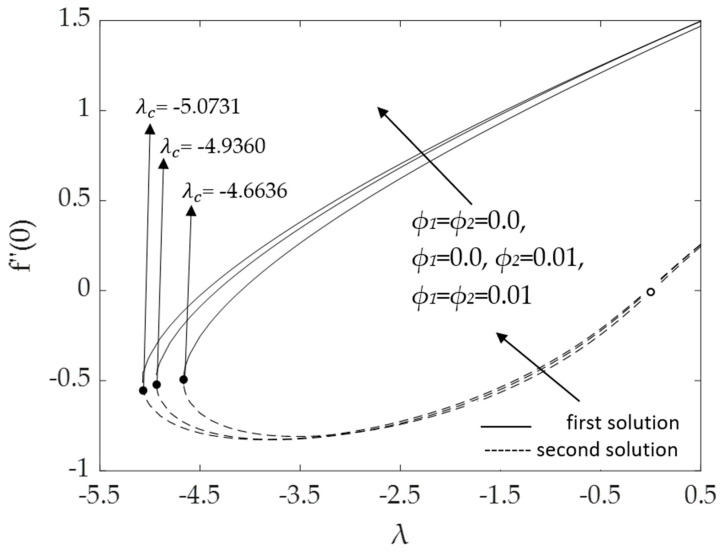
Deviations of $f^{\prime \prime }(0)$ for distinct values of *ϕ*_1_, *ϕ*_2_.

**Figure 5:**
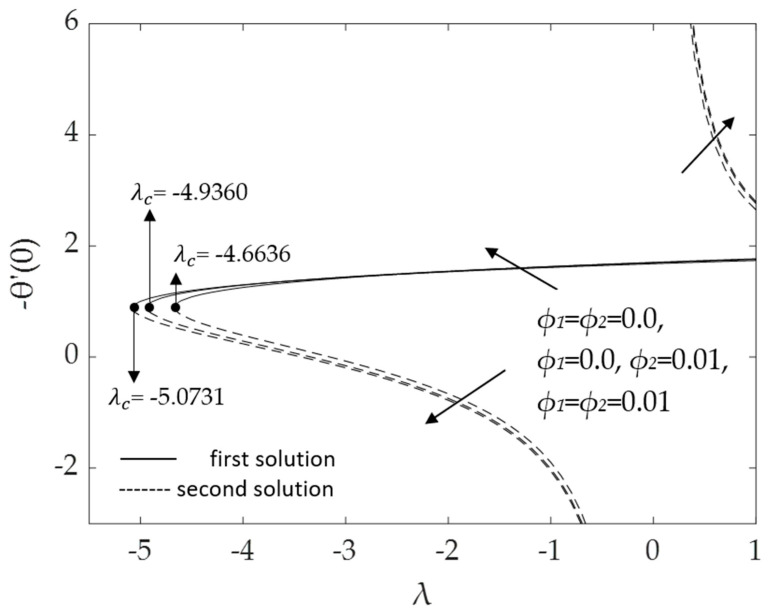
Deviations of $-\theta^{\prime }(0)$ for distinct values of *ϕ*_1_, *ϕ*_2_.

**Figure 6:**
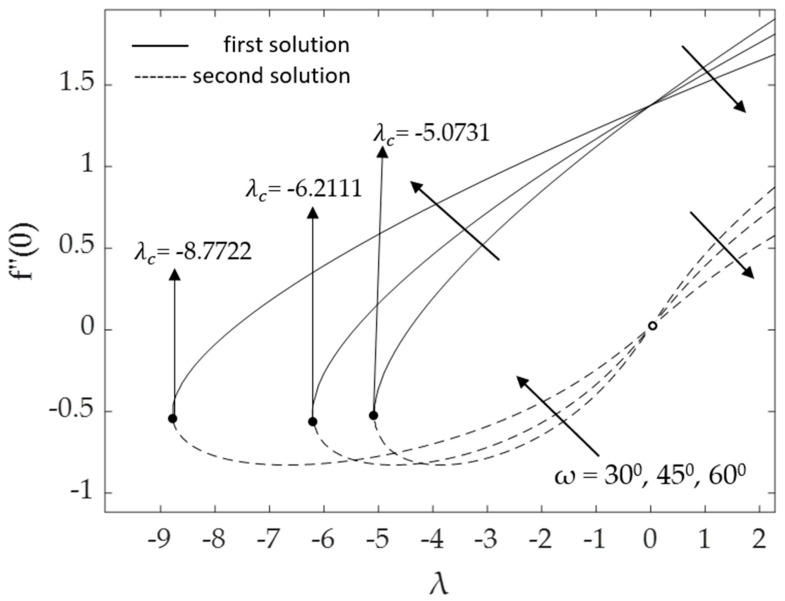
Deviations of $f^{\prime \prime }(0)$ for distinct values of ω.

**Figure 7:**
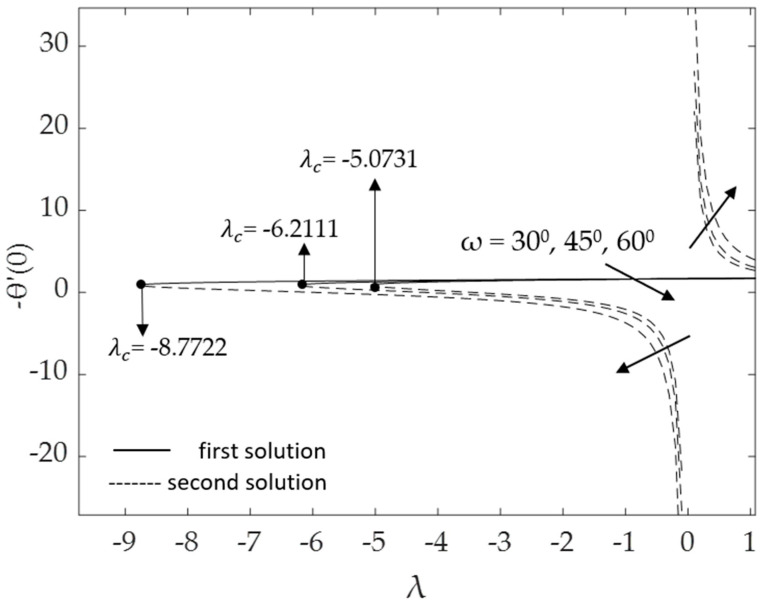
Deviations of $-\theta^{\prime }(0)$ for some values of *ω*.

**Figure 8:**
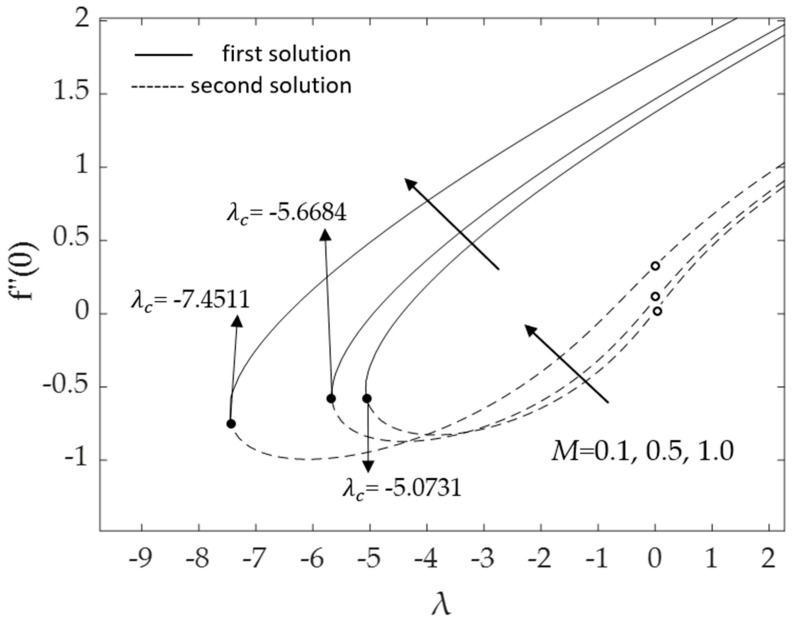
Deviations of $f^{\prime \prime }(0)$ for some values of *M*.

**Figure 9:**
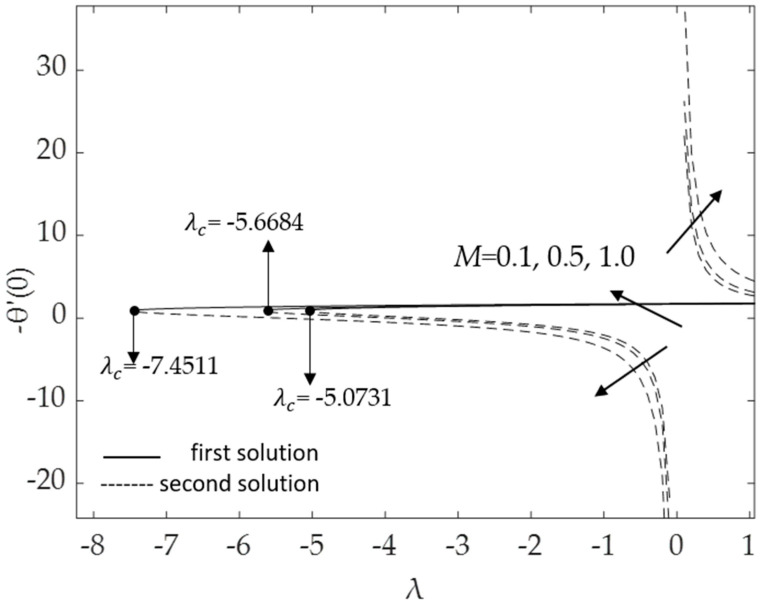
Deviations of $-\theta^{\prime }(0)$ for some values of *M*.

Figure 2, Figure 3, Figure 4, Figure 5, Figure 6, Figure 7, Figure 8 and Figure 9 represent the deviations of the drag forces or the skin friction coefficient and the heat transfer rate for *λ* < 0 (opposing flow) and *λ* > 0 (assisting flows) with mixed convection parameter *λ* for several values of the non-dimensional parameters. Dual solutions are possible for both assisting and opposing flows, as shown in the figures, but the solution is unique for the case of forced convection flow (*λ* = 0). Two solutions exist in the range *λ* > *λ_c_*, and a unique solution is seen at *λ* = *λ_c_*, but there is no solution when *λ* < *λ_c_*. Here, *λ_c_* denotes the bifurcation point or the critical value of *λ* for which the solutions are in existence. This critical value *λ_c_* is a unique value where the first solution meets the second solution, after which the boundary layer separates from the surface, and no solution is found using the boundary layer approximations.

Figure 2 and Figure 3, respectively, show the effects of the curvature parameter *K* on the skin friction coefficient and the local Nusselt number (heat transfer rate at the surface). The figures illustrate that when the curvature parameter *K* increases from *K* = 0 (the surface of the cylinder behaves as a flat surface) to cylinders (*K* = 0.2, 0.4), both $f^{\prime \prime }(0)$ and $-\theta^{\prime }(0)$ increase for the first solution. This is due to the decrement in the radius of the cylinder and the increment of the fluid motions. Less contact between the fluid and the cylinder surface causes a reduction in resistance to the fluid flow. Moreover, increasing *K* will delay the boundary layer separation. Using cylindrical surfaces in boundary layer problems is one approach to obtain a high skin friction and a better heat transfer rate. However, the second solution demonstrates the contrary, with $f^{\prime \prime }(0)$ values that reduce as *K* rises. The second solution presented in Figure 3 produces the opposite results for both flow regions. The heat transfer rate $-\theta^{\prime }(0)$ increases for the phenomenon of buoyancy assisting flow (*λ* > 0); however, it decreases for the phenomenon of buoyancy opposing flow (*λ* < 0) due to the superior impacts of *K*.

Figure 4 and Figure 5 demonstrate the influences of the hybrid nanoparticle volume fractions *ϕ*_1_, *ϕ*_2_ on the skin friction and heat transfer rate, respectively, when *M* = 0.1, Pr = 6.2, *ω* = 30^0^ and *K* = 0.2. It was discovered that the bifurcation value *λ_c_* for water (*ϕ*_1_ = *ϕ*_2_ = 0.0) was −4.6636; for Cu/H_2_O nanofluid (*ϕ*_1_ = 0.0, *ϕ*_2_ = 0.01) it was −4.9360; and for Al_2_O_3_-Cu/H_2_O hybrid nanofluid (*ϕ*_1_ = *ϕ*_2_ = 0.01), it was −5.0731. It was realized that the bifurcation values *λ_c_* (in the opposing region) and the values of $f^{\prime \prime }(0)$ increase when the hybrid nanoparticle volume fractions increase. The results prove that the augmentation of the hybrid nanoparticle volume fraction improves fluid viscosity and thus enhances skin friction, leading to an increase in drag forces. Figure 5 illustrates the same dual solutions pattern as Figure 3. As the nanoparticle volume fractions ϕ_1_, *ϕ*_2_ increase, the heat transfer rate for the first solution also increases, which implies that the Al_2_O_3_-Cu/H_2_O hybrid nanofluid transfers heat more efficiently than the Cu/H_2_O nanofluid and pure base fluid. However, for the second solution, the heat transfer rate decreases for the opposing flow but rises for the assisting flow. It is worth noting that a hybrid nanofluid can have a higher heat transfer rate than a regular nanofluid as well as a pure fluid, as has been proved experimentally and numerically by numerous researchers, see for example [52,53].

Moreover, Figure 6 and Figure 7 display the changes in the values of the skin friction coefficient and the heat transfer rate of the dual solutions for the numerous values of an inclination angle *ω* when Pr = 6.2, *M* = 0.1, *ϕ*_1_ = *ϕ*_2_ = 0.01 and *K* = 0.2, respectively. The value of $f^{\prime \prime }(0)$ for opposing flow (*λ* < 0) increases as the inclination angle rises. Physically, as the value of *ω* increases relative to the posited *x*-axis and when the curvature parameter is present, the effect of buoyancy force due to gravity decreases, causing $f^{\prime \prime }(0)$ to increase but $-\theta^{\prime }(0)$ to decline. The critical values *λ_c_* = −5.0731, −6.2111, and −8.7722 for *ω =* 30°, 45°, and 60°, respectively, are shown in this illustration as well. The larger values of inclination angle augment the drag force, thus lowering the velocity within the boundary layer. The reason for this is the decreasing effect of gravity (see Rehman et al. [54]). Figure 7 illustrates how the increasing value of the inclination angle reduces the rate of heat transmission. As the temperature rises, the rate of heat transmission decreases. The influences of the drag force and heat transfer rate against *λ* for some values of magnetic parameter *M* when *ω* = 30°, Pr = 6.2, *K* = 0.2 and *ϕ*_1_ = *ϕ*_2_ = 0.01 are demonstrated in Figure 8 and Figure 9. Because of the Lorentz force, the velocity and temperature gradients escalate with rising values of the magnetic parameter. This force is generated by the presence of a magnetic field in the fluid flow, which forms resistance to the movement of the fluid. As a result, the skin friction increases, raising the velocity gradient $f^{\prime \prime }(0)$ and the temperature gradient $-\theta^{\prime }(0)$, which are proportional to the skin friction and the heat transfer rate, respectively (see Equation (12)). In addition, in the presence of a magnetic field (*M* > 0), the heat transfer rate is enhanced as the inclination angle increases. This is because the magnetic force counteracts the gravitational buoyancy force at <90° but no longer counteracts it at *ω* = 90°.

**Figure 10:**
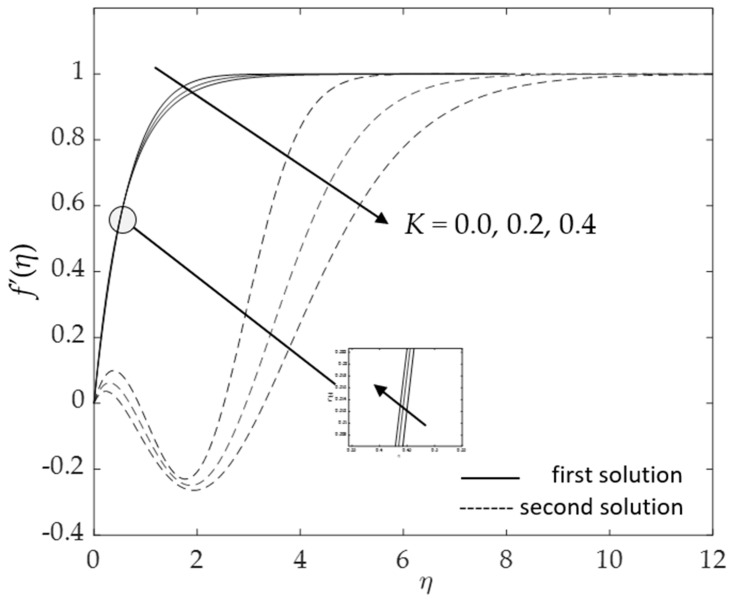
Influence of *K* on $f^{\prime }(\eta )$ for assisting flow.

**Figure 11:**
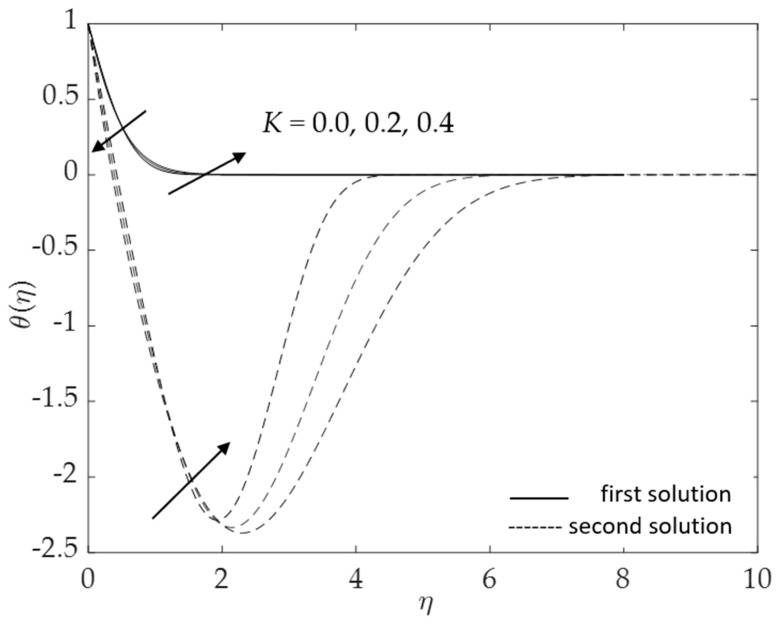
Influence of *K* on *θ*(*η*) for assisting flow.

**Figure 12:**
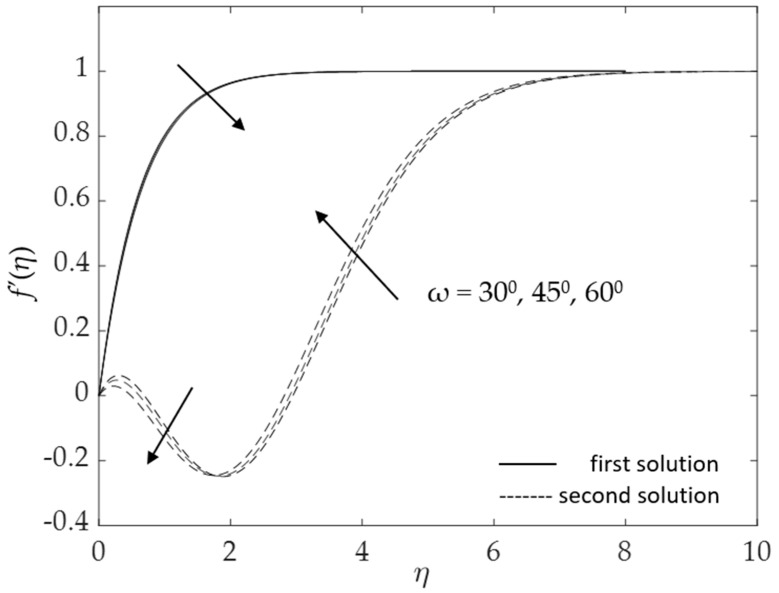
Influence of *ω* on $f^{\prime }(\eta )$ for assisting flow.

**Figure 13:**
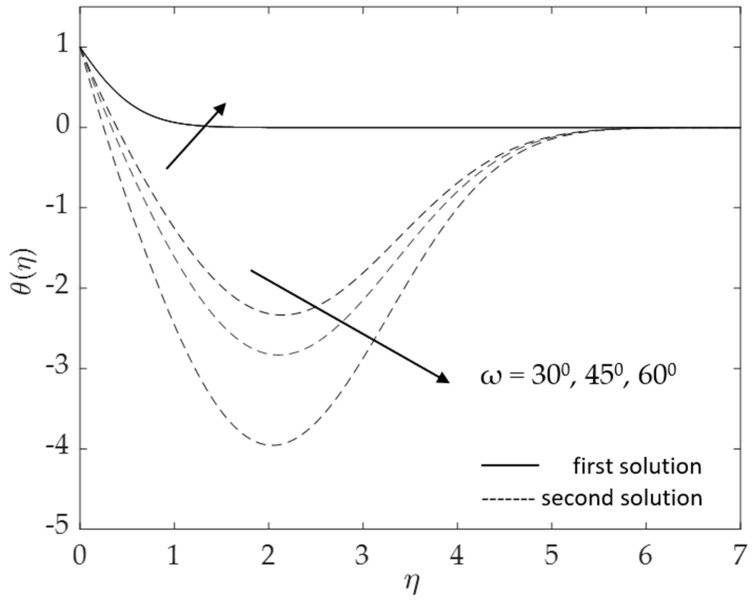
Influence of *ω* on *θ*(*η*) for assisting flow.

**Figure 14:**
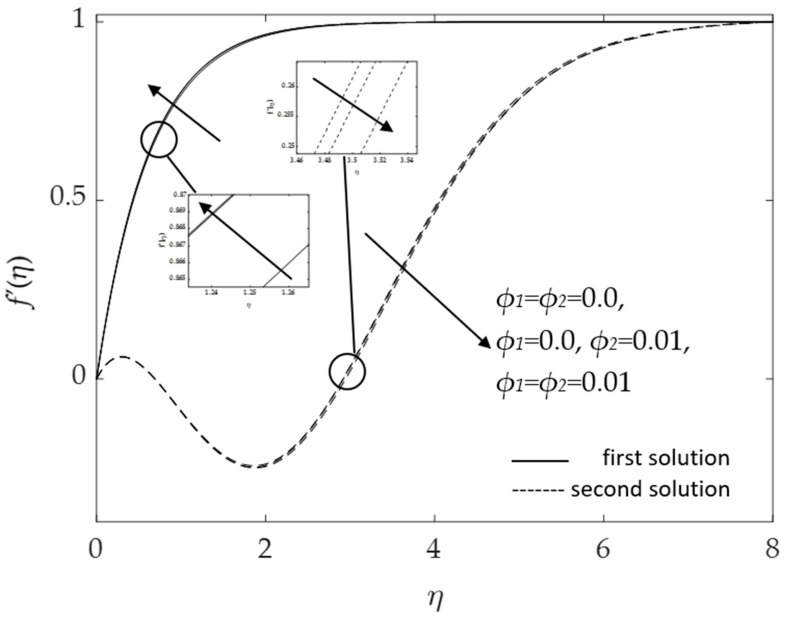
Influence of *ϕ*_1_, *ϕ*_2_ on $f^{\prime }(\eta )$ for assisting flow.

**Figure 15:**
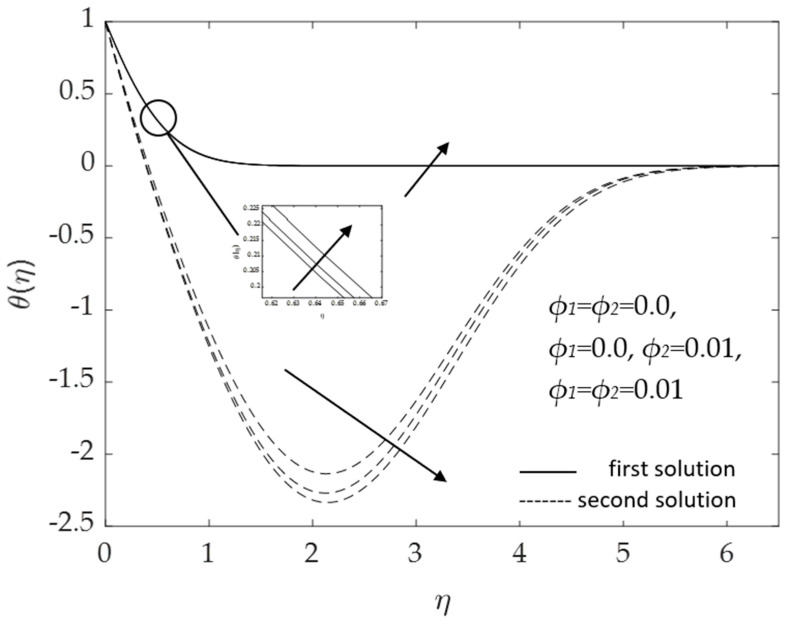
Influence of *ϕ*_1_, *ϕ*_2_ on *θ*(*η*) for assisting flow.

**Figure 16:**
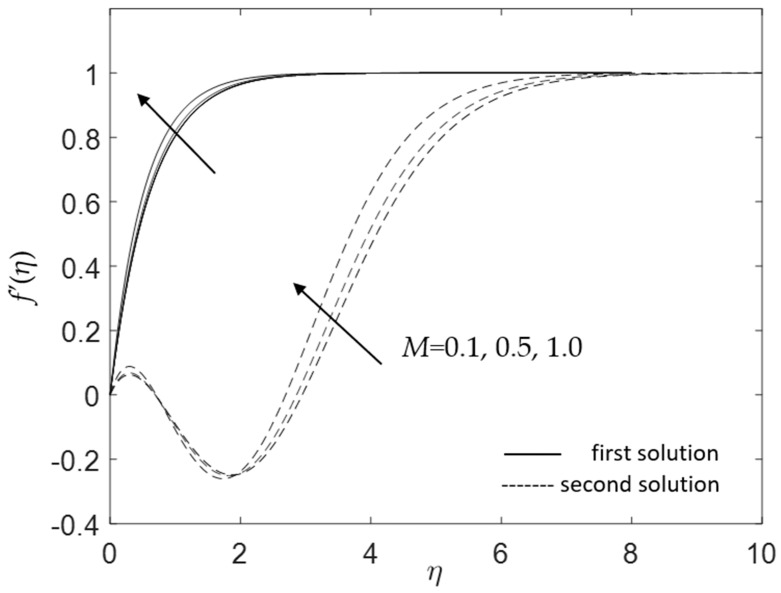
Influence of *M* on $f^{\prime }(\eta )$ for assisting flow.

**Figure 17:**
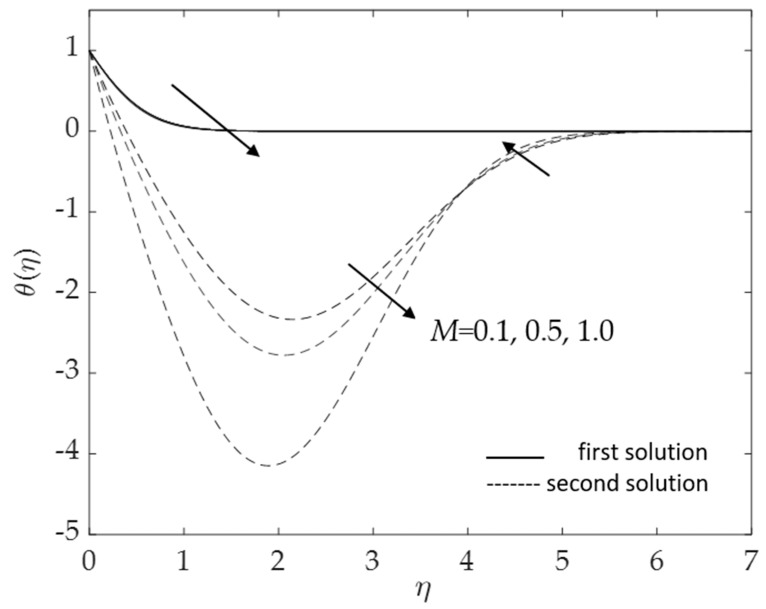
Influence of *M* on *θ*(*η*) for assisting flow.

**Figure 18:**
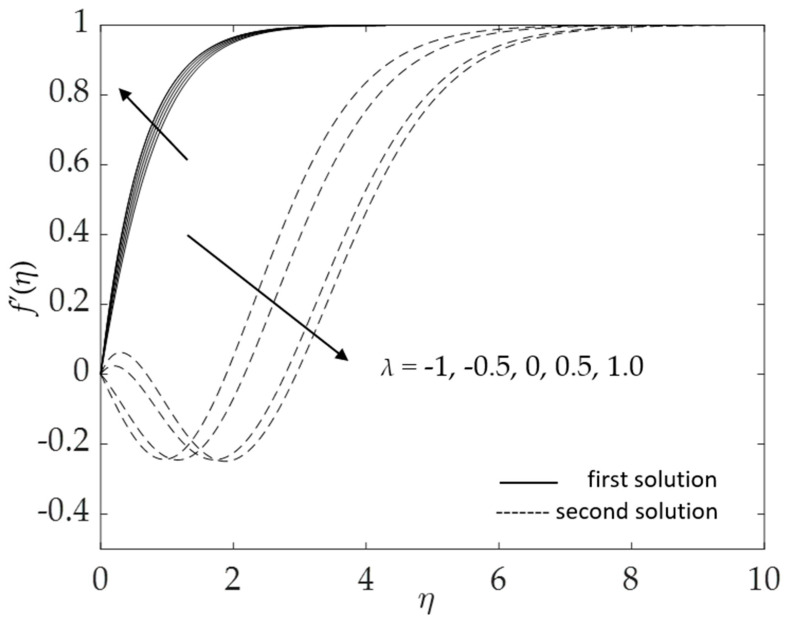
Influence of *λ* on $f^{\prime }(\eta )$.

**Figure 19:**
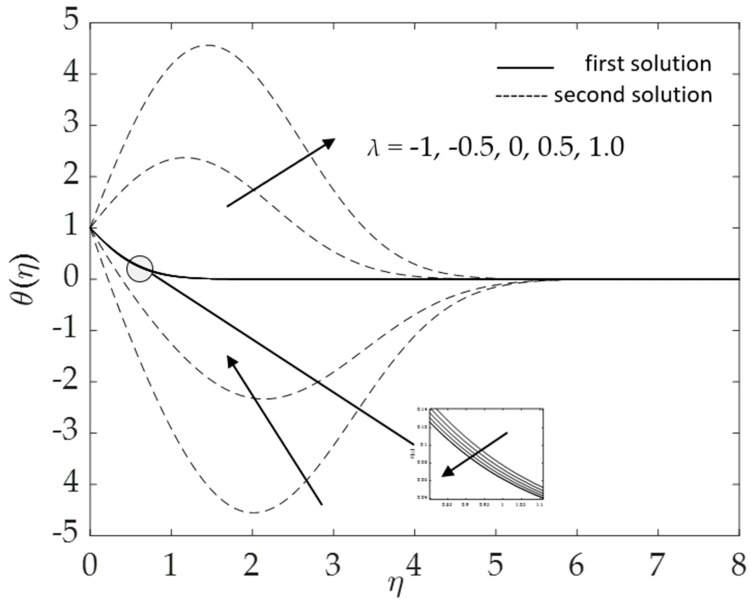
Influence of *λ* on *θ*(*η*).

Figure 10, Figure 11, Figure 12, Figure 13, Figure 14, Figure 15, Figure 16, Figure 17, Figure 18 and Figure 19 reveal the variations in velocity and temperature profiles for assisting flow (*λ* > 0) with several values of non-dimensional parameters. Figure 10 and Figure 11 demonstrate the influences of *K* on the fluid velocity as well as the fluid temperature. At first, $f^{\prime }(\eta )$ increases when the curvature parameter increases. This tendency is seen because of the larger impacts of the curvature parameter; the more it decreases the radius of the cylinder and then accelerates the fluid motions, the less contact there is with the cylinder surface, which causes a reduction in resistance to fluid flow. However, the opposite effect is seen when $\eta_{\infty }$ is approximately greater than one. In contrast, the temperature field shows the opposite behavior to the curves of velocity. The temperature initially decreases ($\eta_{\infty }<1$) and then rises when $\eta_{\infty }$ is roughly greater than one.

Further, the effects of the inclination angle *ω* on the profiles are depicted in Figure 12 and Figure 13. Physically, an increase in the inclination angle reduces the buoyancy effects, thus decreasing the velocity but enhancing the temperature in the boundary layers. The results in these figures correspond to the results shown in Figure 6 and Figure 7. As can be seen in Figure 12, when the inclination angle rises, the velocity decreases (for both solutions). However, the second solution augments when the boundary layer thickness $\eta_{\infty }$ is roughly greater than two. Meanwhile, Figure 13 shows a similar trend where the second solution displays the opposite behavior. We note that in the present study, there is a reduction to a vertical cylinder when *ω* = 0° and to a horizontal cylinder, when *ω* = 90°. The buoyancy effects for the latter case are very small and thus can be neglected.

Figure 14 and Figure 15 portray the influences of the hybrid nanoparticle volume fraction *ϕ*_1_, *ϕ*_2_ for the base fluid (*ϕ*_1_ = *ϕ*_2_ = 0.0), Cu/H_2_O nanofluid (*ϕ*_1_ = 0.0, *ϕ*_2_ = 0.01) and Al_2_O_3_-Cu/H_2_O hybrid nanofluid (*ϕ*_1_ = *ϕ*_2_ = 0.01). It was found that the velocity elevates when the volume of the nanoparticles augments, as presented in Figure 14. The thickness of the momentum boundary layer reduces as the concentration of nanoparticles uplifts. Hence, the velocity of the fluid flow increases. Figure 15 shows that as the hybrid nanoparticle rises, the temperature curves and the thermal boundary layer thickness also upsurges. The surface heat flux lowers as the thermal boundary layer thickens. These discoveries are according with the results illustrated in Figure 4 and Figure 5. However, the second solutions for both figures show contradictory behavior, which is associated with the assisting flow (*λ* = 1).

Apart from that, Figure 16 and Figure 17 present the effects of the magnetic parameter *M* on the velocity and temperature profiles when *ω* = 30°, Pr = 6.2, *K* = 0.2, *ϕ*_1_ = *ϕ*_2_ = 0.01 and *λ* = 1. By increasing the values of *M*, the velocity increases, as portrayed in Figure 16. The momentum boundary layer becomes thinner as the impact of *M* is boosted, causing an increase in the velocity gradient at the surface $f^{\prime \prime }(0)$. Thus, the skin friction coefficient increases with increasing *M* for the present case. This result is consistent with that shown in Figure 8. Additionally, Figure 17 shows that the temperature distribution reduces as the magnetic parameter rises. Deceleration in the thermal boundary layer thickness leads to the increment of the heat flux. This shows a similar trend to that shown in Figure 15. This finding agrees with the illustration in Figure 9.

Figure 18 and Figure 19 show the influences of the buoyancy force on the velocity and temperature for numerous values of the mixed convection parameter *λ* when *ω* = 30°, Pr = 6.2, *K* = 0.2, *ϕ*_1_ = *ϕ*_2_ = 0.01 and *M* = 0.1. In Figure 18, the velocity increases for the first solution, whereas the second solution shows the opposite trend with the increment of *λ* (opposing to assisting flow). Nevertheless, the temperature for the first solution decreases when the mixed convection parameter increases, as shown in Figure 19. The second solution presents inconsistent behavior in the temperature profile.

Table 5 presents the smallest eigenvalues, *γ*, of the Al_2_O_3_–Cu/H_2_O hybrid nanofluid for several values of *K* when *M* = 0.1, Pr = 6.2, *ω* = 30° and *ϕ*_1_ = *ϕ*_2_ = 0.01. The obtained minimum eigenvalues are positive for the first solution, but negative for the second solution. Referring to Equation (19), positive values of *γ* indicate a decay of disturbance as time evolves τ→∞. On the other hand, negative values of *γ* show an initial growth in disturbance, and thus the solutions obtained for this case are unstable in the long run. Moreover, for both solutions, the smallest eigenvalue *γ* approaches zero as *λ* approaches the bifurcation value *λ_c_*. This means that, at turning points (from the first to the second solution), there is a change in the values of *λ* from stable (positive) to unstable (negative). Thus, this finding supports the stability and physical reliability of the first branch solutions. As shown by the temporal stability analysis, the second solution is unstable in the long run. Although such solutions may lack physical significance (in the long run), they are still of mathematical interest as far as the differential equations are concerned. The second solution is also a solution to the differential equations. Similar equations may arise in other situations where the corresponding solution can have a more realistic meaning.

## 5. Conclusions

The present study investigated the behavior of the mixed convection flow of an Al_2_O_3_-Cu/H_2_O hybrid nanofluid over an inclined cylindrical surface. The effects of the dimensionless parameters on the skin friction coefficient and the heat transfer rate at the surface were numerically computed and graphically presented. The summarized discoveries are listed as follows:

- Two solutions were obtained for both buoyancy assisting and opposing flows, whereas a unique solution was found in the absence of buoyancy force (*λ* = 0);
- Larger inclination angles *ω* lead to a lower gravitational force, increasing the friction factor $f^{\prime \prime }(0)$ but decreasing the heat transfer $-\theta^{\prime }(0)$;
- The skin friction coefficient and the heat transfer rate of the Al_2_O_3_-Cu/H_2_O hybrid nanofluid (*ϕ*_1_ = *ϕ*_2_ = 0.01) is higher than that of the nanofluid (*ϕ*_1_ *=* 0%, *ϕ*_2_ *=* 1%) and water (*ϕ*_1_ = *ϕ*_2_ = 0.0);
- Larger values of the curvature parameter *K* and mixed convection parameter *λ* result in a slower detachment of the boundary layer;
- Velocity increases with increasing values of the buoyancy parameter *λ*, magnetic parameter *M*, and hybrid nanofluid nanoparticle volume fraction *ϕ*_1_, *ϕ*_2_, whereas it declines for curvature parameter *K* and the inclined angle *ω*;
- The velocity and temperature gradients upsurge with a higher impact of the magnetic parameter;
- According to the temporal stability analysis, the first solution is physically stable as time evolves, whereas the second solution is not reliable in the long run.

## Figures and Tables

**Table 1:** Thermophysical correlations of Al_2_O_3_–Cu/H_2_O (see Takabi and Salehi [40], Ganguly et al. [41] and Waini et al. [42]).

Properties	Hybrid Nanofluid
Density	$\rho_{hnf}=\phi_{1}\rho_{Al}+\phi_{2}\rho_{C}+\left(1-\phi_{hnf}\right)\rho_{f}$
Dynamic viscosity	$\mu_{hnf}=\mu_{f}{\left(1-\phi_{1}-\phi_{2}\right)}^{-2.5}$
Thermal conductivity	$k_{H}=\frac{\;\frac{\phi_{1}k_{Al}+\phi_{2}k_{C}}{\phi_{1}+\phi_{2}}+2k_{f}+2\left(\phi_{1}k_{Al}+\phi_{2}k_{C}\right)-2\left(\phi_{1}+\phi_{2}\right)k_{f}\;}{\frac{\phi_{1}k_{Al}+\phi_{2}k_{C}}{\phi_{1}+\phi_{2}}+2k_{f}-\left(\phi_{1}k_{Al}+\phi_{2}k_{C}\right)+\left(\phi_{1}+\phi_{2}\right)k_{f}}\;$
Electrical conductivity	$\sigma_{H}=\frac{\frac{\phi_{1}\sigma_{Al}+\phi_{2}\sigma_{C}}{\phi_{1}+\phi_{2}}+2\sigma_{f}+2\left(\phi_{2}\sigma_{Al}+\phi_{1}\sigma_{C}\right)-2\left(\phi_{1}+\phi_{2}\right)\sigma_{f}}{\frac{\phi_{1}\sigma_{Al}+\phi_{2}\sigma_{C}}{\phi_{1}+\phi_{2}}+2\sigma_{f}-\left(\phi_{1}\sigma_{Al}+\phi_{2}\sigma_{C}\right)+\left(\phi_{1}+\phi_{2}\right)\sigma_{f}}$
Heat capacity	${\left(\rho C_{p}\right)}_{hnf}=\left(1-\phi_{hnf}\right){\left(\rho C_{p}\right)}_{f}+\phi_{1}{\left(\rho C_{p}\right)}_{Al}+\phi_{2}{\left(\rho C_{p}\right)}_{C}$
Thermal expansion	${\left(\rho \beta \right)}_{hnf}=\left(1-\phi_{hnf}\right){\left(\rho \beta \right)}_{f}+\phi_{1}{\left(\rho \beta \right)}_{Al}+\phi_{2}{\left(\rho \beta \right)}_{C}$

**Table 2:** Thermophysical characteristics of the regular fluid and hybrid nanoparticles (see Waini et al. [42] and Oztop and Abu-Nada [43]).

Physical Properties	Water	Cu	Al_2_O_3_
$C_{p}\left(J/KgK\right)$	4179	385	765
$\beta \times 10^{-5}\left(K^{-1}\right)$	21	1.67	0.85
$k\left(W/mK\right)$	0.613	400	40
$\sigma \left(S/m\right)$	0.05	$5.96\times 10^{7}$	$3.69\times 10^{7}$
$\rho \left(kg/m^{3}\right)$	997.1	8933	3970
Pr	6.2		

**Table 3:** The output values of the skin friction coefficient for several values of Pr.

Pr	Lok et al. [51] (Keller-Box)	Ishak et al. [28] (Keller-Box)		Current Study (bvp4c)	
	First Solution	First Solution	Second Solution	First Solution	Second Solution
0.7	1.706376	1.7063	1.2387	1.706323	1.238728
1.0	-	1.6755	1.1332	1.675437	1.133192
7.0	1.517952	1.5179	0.5824	1.517913	0.582401
10.0	-	1.4928	0.4958	1.492839	0.495779
20.0	1.448520	1.4485	0.3436	1.448483	0.343640
40.0	1.410094	1.4101	0.2111	1.410058	0.211101
50.0	-	1.3989	0.1720	1.398930	0.172048
60.0	1.390311	1.3903	0.1413	1.390274	0.141292

**Table 4:** The output values of the local Nusselt number for several values of Pr.

Pr	Lok et al. [51] (Keller-Box)	Ishak et al. [28] (Keller-Box)		Current Study (bvp4c)	
	First Solution	First Solution	Second Solution	First Solution	Second Solution
0.7	0.764087	0.7641	1.0226	0.764063	1.022631
1.0	-	0.8708	1.1691	0.870779	1.169126
7.0	1.722775	1.7224	2.2191	1.722382	2.219194
10.0	-	1.9446	2.4940	1.944617	2.494029
20.0	2.458836	2.4576	3.1646	2.457590	3.164608
40.0	3.103703	3.1011	4.1080	3.101093	4.108024
50.0	-	3.3415	4.4976	3.341458	4.497588
60.0	3.555404	3.5514	4.8572	3.551406	4.857187

**Table 5:** The eigenvalue *γ* for distinct values of *K* for Al_2_O_3_–Cu/H_2_O when *M* = 0.1, Pr = 6.2, *ω* = 30° and *ϕ*_1_ = *ϕ*_2_ = 0.01.

K	λ	Smallest Eigenvalue	
		First Solution	Second Solution
0.0	−4.5	0.0972	−0.0963
	−4.51	0.0641	−0.0641
	−4.517	0.0199	−0.0218
	−4.5177	0.0104	−0.0103
0.2	−4.5	0.5486	−0.5091
	−5.0	0.1996	−0.1943
	−5.07	0.0709	−0.0703
	−5.073	0.0597	−0.0592
	−5.0731	0.0593	−0.0588
	−4.5	0.7357	−0.6675
0.4	−5.0	0.5353	−0.4991
	−5.5	0.2065	−0.2010
	−5.55	0.1385	−0.1361
	−5.552	0.1351	−0.1328
	−5.5521	0.1350	−0.1326

## Data Availability

Not applicable.
